# Transient and Flexible Hyperbolic Metamaterials on Freeform Surfaces

**DOI:** 10.1038/s41598-018-27812-4

**Published:** 2018-06-21

**Authors:** Hung-I Lin, Kun-Ching Shen, Shih-Yao Lin, Golam Haider, Yao-Hsuan Li, Shu-Wei Chang, Yang-Fang Chen

**Affiliations:** 1 Graduate Institute of Applied Physics, National Taiwan University, Taipei, 106 Taiwan; 2 Department of Physics, National Taiwan University, Taipei, 106 Taiwan; 3 Research Center for Applied Sciences, Academia Sinica, Taipei, 115 Taiwan

## Abstract

Transient technology is deemed as a paramount breakthrough for its particular functionality that can be implemented at a specific time and then totally dissolved. Hyperbolic metamaterials (HMMs) with high wave-vector modes for negative refraction or with high photonic density of states to robustly enhance the quantum transformation efficiency represent one of the emerging key elements for generating not-yet realized optoelectronics devices. However, HMMs has not been explored for implementing in transient technology. Here we show the first attempt to integrate transient technology with HMMs, *i.e*., transient HMMs, composed of multilayers of water-soluble and bio-compatible polymer and metal. We demonstrate that our newly designed transient HMMs can also possess high-*k* modes and high photonic density of states, which enables to dramatically enhance the light emitter covered on top of HMMs. We show that these transient HMMs devices loss their functionalities after immersing into deionized water within 5 min. Moreover, when the transient HMMs are integrated with a flexible substrate, the device exhibits an excellent mechanical stability for more than 3000 bending cycles. We anticipate that the transient HMMs developed here can serve as a versatile platform to advance transient technology for a wide range of application, including solid state lighting, optical communication, and wearable optoelectronic devices, etc.

## Introduction

Transient technology is foreseeable to be a key ingredient for the development of emerging devices, especially for the sake of a sustainable human society^1,2^. Scientists exploit materials, devices and systems that are capable of completely being dissolved or selectively disintegrated under controlled manners at prescribed times^3–6^. Since no toxic products are released, they can be used to replace some existing devices with environmentally friendly alternatives which disintegrates when exposed to water. Potential applications span from military uses, consumer optoelectronics and even to bio-medical implants^1,7–9^. Importantly, to track or recycle every gadget (*e.g*., hidden cameras, miniature sensors, confidential chips and artificial intelligence robotics) that leaves or crashes in a war zone is nearly impossible creating enticing opportunities for adversaries to collect, study, reverse-engineer and even copy. Thus, people expect next generation military hardware or device with sensitive information can literally cease to exist as soon as mission completed to prevent the critical loss of intellectual properties and technological advances. In addition, consumer optoelectronics, including portable electronics (*e.g*., smartphones, tablets and laptops) and environmental sensors (*e.g*., photo/gas/thermal/pressure detectors) together with their network for Internet of Things (IoT), have greatly promoted qualities of life^10^. However, rapid technological advances have led to a significant decrease in the lifetime of non-degradable semiconductors based consumer electronics that approaching an average of several months to few years. The hope of sustainable society therefore goes on transient modules. For consumer products can be decomposed for days rather than trash. Furthermore, the growing requirements of healthcare quality have sparked an emerging research trend in transient bio-medical components^5,11^. Recent advances can be classified into two types: one is designed for long-term uses to avoid reiterative surgeries for therapeutic device replacement (*e.g*., brain electrical stimulators, cardiac pacemakers, *in vivo* nanogenerator/battery and bio-interfaced systems for real time point-of-care diagnostics)^12–14^; another is realized as temporary medical tasks in the human body after they provide some useful function (*e.g*., localized drug delivery, injectable electrophysiological signal monitor and digital imaging sensors)^11–13,15^.

Until recently, another key research highlight is controlling the flow of electromagnetic wave by shaping its phase, amplitude and polarization, for expanding its diversity to achieve paramount breakthroughs^16–18^. Such promising advances have been successfully accomplished in a variety of applications, *e.g*., metamaterials^18,19^, negative refractive index, epsilon-near zero (ENZ)^20^, Cherenkov radiation^21^ and increasing nonlinear optical response^18,22^. Especially, ENZ metamaterials can squeeze the electromagnetic wave through a narrow waveguide channels, resulting in the potential applications for sensing, focusing and collimating, filtering and controlling light directivity in the visible region^23–25^. Furthermore, one of the widespread research topics is hyperbolic metamaterials (HMMs), defined by the iso-frequency curve in momentum-space owing to its unique hyperbolic shape instead of ellipse for the common dielectric materials^26–29^. To achieve this artificial structure with promising functionality that provides high-*k* modes of the unbounded wave-vector from the increased photonic density of states (PDOS), proper design of metal-dielectric multilayers with alternated composition or well-aligned metallic nanowires embedded in a dielectric media has been proven to be very useful^26^. This unique characteristic makes HMMs beneficial for such as highly sensitive bio-sensors to detect ultralow-molecular-weight (244 Da) bio-molecules for extremely diluted concentrations (*e.g*., 10 femto-molar) to reach a record figure of merit (FOM) of 590 based on the exciting of high-*k* modes^30^. For the biology, photonics, optoelectronics and chemistry applications, HMMs have been proved to increase the charge separation for 2.4 times and recombination rate for 1.7 times as confirmed by Marcus theory^31^. From the PDOS aspect, HMMs with nano-patterned structures are able to out-coupled the energy, resulting in an 80-fold enhancement of the spontaneous emission dynamics^32^. In addition, these nano-patterned structures can approach the physical limit with a sufficient out-coupled effect of photoelectric transformation efficiency^33,34^. Besides, the cylindrical HMMs nano-antenna structures can enhance the emitted power into free-space for 100-fold^35^. As to the application of stimulated emission (*e.g*., laser), HMMs can also enhance the transition rate of the optical gain media to achieve the lasing action easily and reduce the threshold, which is very useful to serve as a suitable candidate for all-optical communication^36–39^. Moreover, it is theoretically suggested that HMMs can play a significant role for invisible cloaking and low-scattering near-field optical microscopes in the visible wavelength^40^. Furthermore, by controlling the dispersion engineering from HMMs, it enables preferential power extraction from the desired direction for future high-speed optical transmission applications^41^.

As a proof of concept, here we demonstrate the first attempt of the polymer-based transient HMMs composed multilayers of gold (Au) and poly(vinyl alcohol) (PVA) with water-soluble characteristics. Different thickness compositions show unique features at different wavelengths (*i.e*., high-*k* modes from iso-frequency curves and high PDOS from Purcell factors) of the HMMs as confirmed by the simulation results using three-dimensional (3D) finite-difference time-domain (FDTD) and experimental measurements from the enhanced photoluminescence emission of dye molecules. These transient HMMs devices can be easily washed away and then disappear by just using some drops of deionized (DI) water at room temperature. Within 5 min, the dissolving process can destroy the properties of hyperbolic dispersion, and then after 1.5 hr, the whole transient HMMs device is almost disintegrated. Interestingly, when introducing the concept of transient, a wide variety applications and unexplored functions can be imagined, such as being an invisible cloak for a while but dissolved away in the next minute; implantable medical media as an *in vivo* short-lived bio-sensor in the human body that prevents the long-term side effects; and as consumer electronics for enhancing light emission or as a highly efficient ultra-thin metalens that disappears after use to minimize the environmental damage. Moreover, the transient HMMs are promised to be novel candidates as the high-speed optical modulator temporarily, but disappear after the transmission of confidential files, as well as the nano-antenna to enhance radiative emission from a quantum emitter but eventually loss its functionality after delivering the power. Consequently, this demonstration presents a platform concept for expanding both the research interests of transient technology and metamaterials.

## Results and Discussion

### Design and characteristics of the transient HMMs

**Figure 1:**
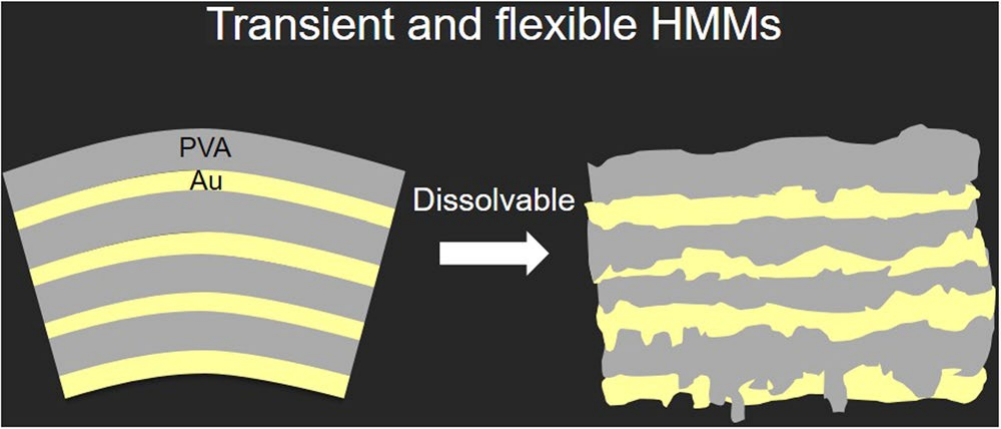
Proof of concept design of transient and flexible hyperbolic metamaterials (HMMs). Schematic diagram of transient and flexible HMMs is illustrated.

Basically, the signs of effective permittivity (ε) or permeability (μ) of the HMMs show the opposite directions in their optical tensors (*i.e*., ${\varepsilon }_{\perp }\cdot {\varepsilon }_{\parallel } < 0$ or ${{\rm{\mu }}}_{\perp }\cdot {{\rm{\mu }}}_{\parallel } < 0$), leading to the hyperbolic dispersion:

1 $$\frac{{{\rm{\omega }}}^{2}}{{c}^{2}}=\frac{{k}_{x}^{2}+{k}_{y}^{2}}{{{\rm{\varepsilon }}}_{\perp }}+\frac{{k}_{z}^{2}}{{{\rm{\varepsilon }}}_{\parallel }},$$

where *c* = speed of light in vacuum^26^. The subscripts of ⊥ and $\parallel$ are the directions perpendicular and parallel to the anisotropy axis, respectively. Therefore, with the proper design of multilayer structures, the excitation of the high-*k* modes and then the extraction of the power out-coupled to the free space can be achieved. Figure 1 illustrates a schematic demonstration platform with utilizing Au/PVA-based transient and flexible HMMs device. We design two fill-fractions by varying Au thickness: 37.31% (marked as the HMM1) and 26.87% (marked as the HMM2), for several dye molecules with different emission wavelengths, including rhodamine 110 (R110), R6G and 4-(dicyanomethylene)-2-*tert*-butyl-6-(1,1,7,7-tetramethyljulolidin-4-yl-vinyl)-4*H*-pyran (DCJTB) dye molecules, and their emission wavelength centers are at 530, 560 and 650 nm, respectively. These dye molecules are embedded inside poly(methyl methacrylate) (PMMA).

### Optical properties of the transient HMMs

**Figure 2:**
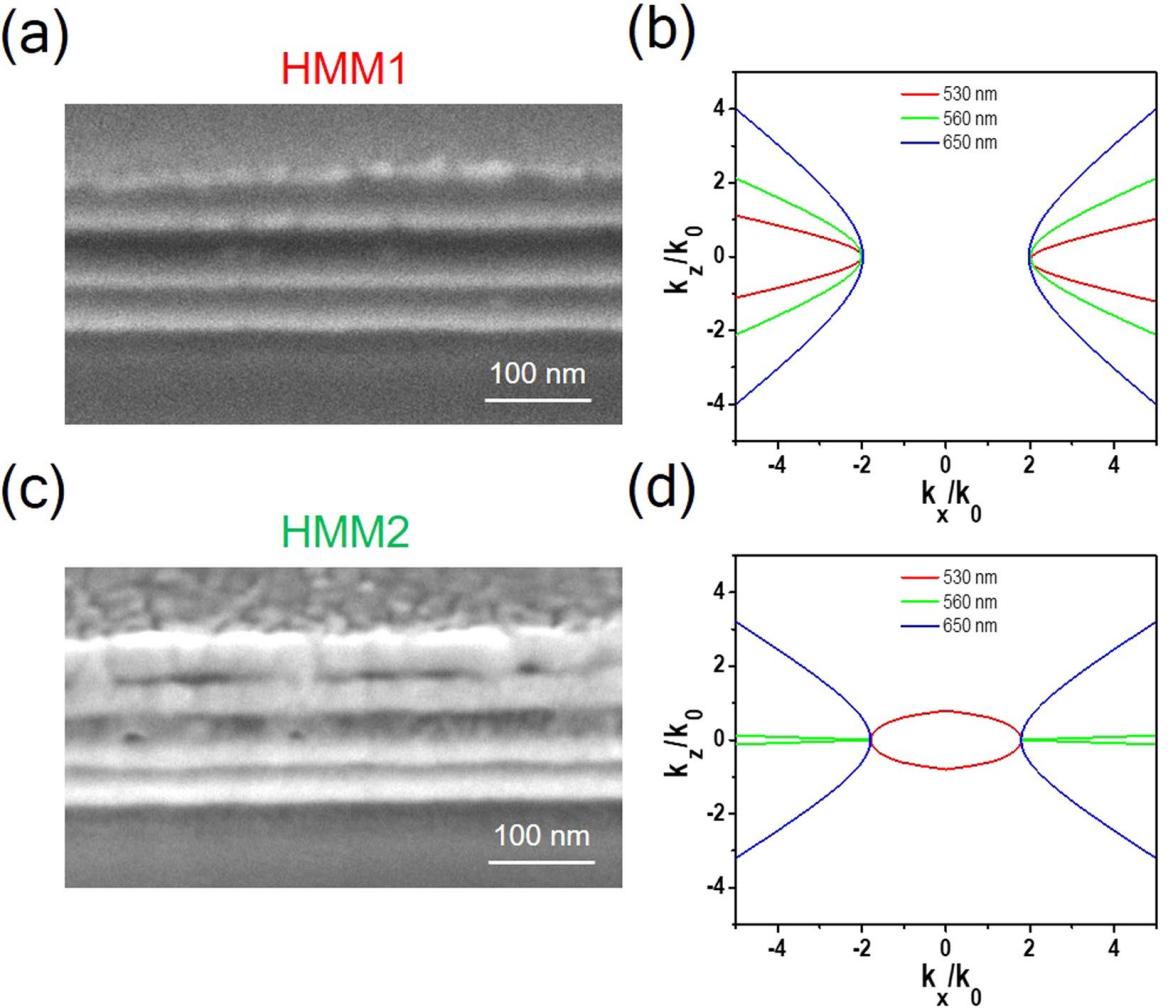
Properties of the transient HMMs. (**a**) is the cross-sectional field emission scanning electron microscopy (FE-SEM) image of the HMM1 with 4 pairs of gold (Au)/poly(vinyl alcohol) (PVA) with thicknesses of 25/42 nm. (**b**) is the iso-frequency curves performed at three wavelengths (*e.g*., 530, 560 and 650 nm) for the HMM1 sample. (**c**) and (**d**) are the FE-SEM image of the HMM2 sample with thicknesses of Au/PVA for 18/49 nm and its corresponding iso-frequency curves, respectively.

To verify the hyperbolic dispersion of our proposed layered structures (for occurring ${{\rm{\varepsilon }}}_{\perp }\cdot {{\rm{\varepsilon }}}_{\parallel } < 0$), we calculate ${\rm{Re}}({{\rm{\varepsilon }}}_{\perp })$ and ${\rm{Re}}({{\rm{\varepsilon }}}_{\parallel })$ based on the effective medium theory (EMT) of the HMM1 (HMM2) with the corresponding wavelength of 520 (560) nm as shown in Fig. S1a,b). The energy of incident light can be delivered inside the layered structure owing to the hyperbolic dispersion (*i.e*., the existence of high-*k* modes)^42^. With considering the HMM1 (HMM2), the multilayer thicknesses of Au/PVA are 25/42 (18/49) nm. Cross-sectional field emission scanning electron microscope (FE-SEM) image of the HMM1 and its corresponding iso-frequency curves performed at three different wavelengths (*e.g*., 530, 560 and 650 nm) are shown in Fig. 2a,b, respectively. Clear multilayer structures with flat thin film can be observed, providing the underlying structure for the allowed propagation of the high-*k* modes inside the multilayer structures. Furthermore, the FE-SEM image of the HMM2 and its iso-frequency curves are presented in Fig. 2c,d, respectively. We designed the HMM2 sample at the wavelength of 530 nm showing the fact that the dispersion curve is elliptic (marked as the EMMs) as a comparison to profoundly stand out the importance of the HMMs. We further discuss the out-coupling configurations in the supporting information as shown in Fig. S2.

Figure S3 presents the calculation steps of the effective permittivities originated from the optical properties (*i.e*., transmission and reflection) of our proposed transient HMMs structures^43^. In Fig. S3a,b, the phenomena of strong absorption are achieved (*e.g*., more than 80% in the shorter wavelength region), which is a typical behavior of HMMs. Detailed derivation process is described in the supporting information. Thus, we can obtain the complex refractive index: $(n+{\rm{i}}k)$, for the HMM1 and the HMM2 samples as shown in Fig. S1c,d, respectively. Finally, the effective permittivity:

2 $${{\rm{\varepsilon }}}_{y}^{eff}={(n+{\rm{i}}k)}^{2},$$

is shown in Fig. S3e,f. It is obvious that the reflectance is larger at long wavelength region, which can be ascribed to the smaller *n* as shown in Fig. S1c,d. The largest value of reflectance results from the smallest *n* at long wavelength region (~600–700 nm), while the positions of maximum *n* of the HMM1 and HMM2 are 453.6 and 485.1 nm, respectively. The maximum absorbance as shown in Fig. S3c for the HMM1 corresponds to the maximum *n* due to the nearly matched index between the environment (*e.g*., *n* = 1 for air) and our proposed structures. On the other hand, the maximum absorbance occurs at ~373 nm as shown in Fig. S1d for the HMM2, which can be ascribed to the higher transmission appears at ~500 nm, leading to the lower absorption in that wavelength position.

### Spontaneous emission dynamics

**Figure 3:**
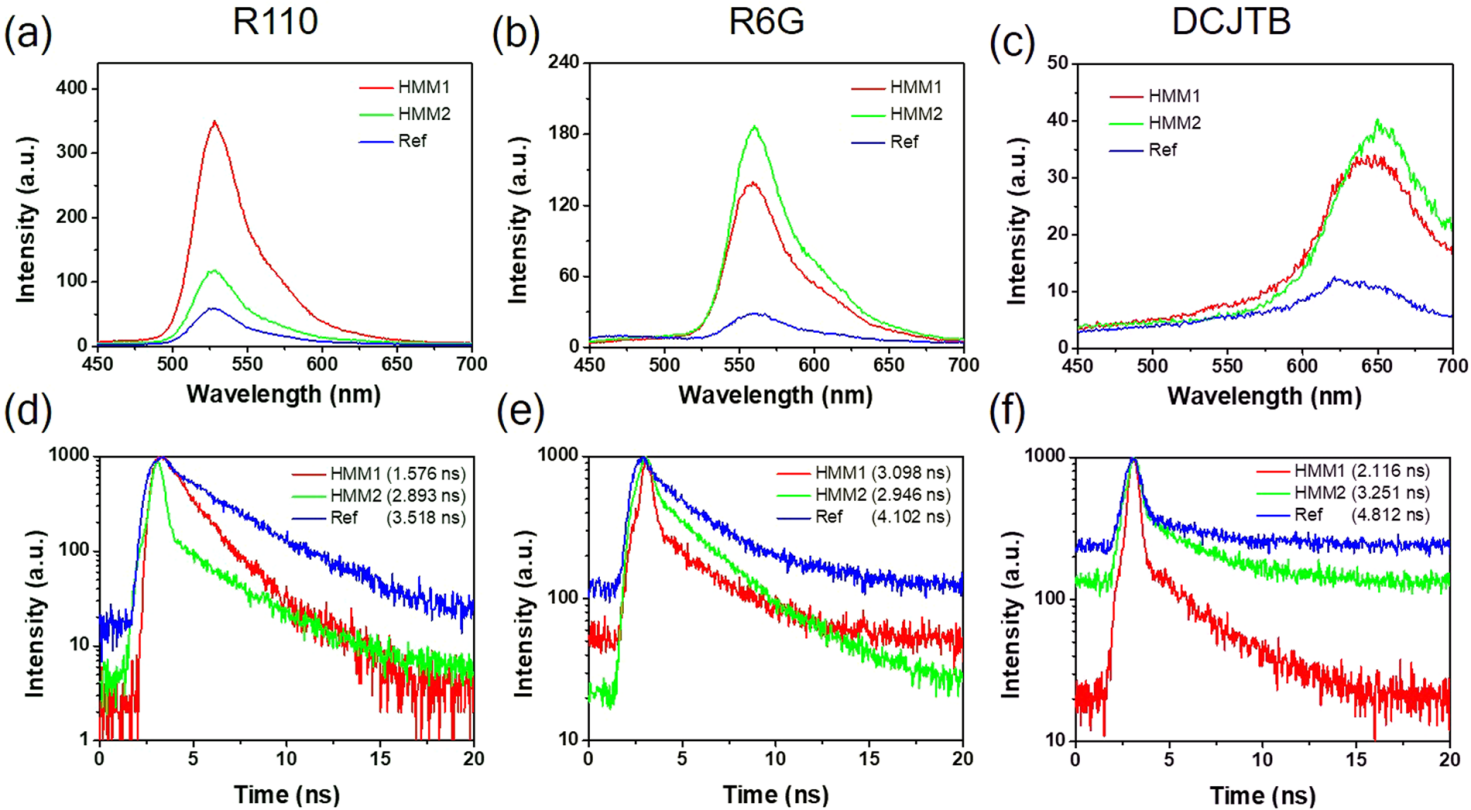
Photoluminescence spectra and the lifetime kinetics. (**a**), (**b**) and (**c**) are the measured photoluminescence spectra using 374 nm pulsed diode laser at the pumping energy density of 103 μJ/cm^2^ for rhodamine 110 (R110), R6G and 4-(dicyanomethylene)-2-*tert*-butyl-6-(1,1,7,7-tetramethyljulolidin-4-yl-vinyl)-4*H*-pyran (DCJTB) dye molecules, respectively. (**d**), (**e**) and (**f**) are the corresponding lifetime measurements.

To explore the spontaneous emission effect arising from the transient HMMs, we measured the photoluminescence kinetics by 374 nm pulsed diode laser at the pumping energy density of 103 μJ/cm^2^ as shown in Fig. 3a–c for R110, R6G and DCJTB dye molecules, respectively. First, for the emission from R110 dye molecule (Fig. 3a), the photoluminescence intensity of the HMM1 (HMM2) sample is 4.94 (0.98) times stronger than the reference sample. The less emission intensity of the HMM2 is attributed to the dispersion of the iso-frequency elliptic curve. Second, for the emission of R6G dye molecule (Fig. 3b), the photoluminescence intensity of the HMM1 (HMM2) sample is 5.55 (3.80) times higher than the reference sample. Third, for the emission of DCJTB dye molecule, the photoluminescence intensity of the HMM1 (HMM2) sample is 2.48 (3.22) times higher than the reference sample (Fig. 3c). Besides, we further performed the time-resolved photoluminescence (TRPL) to realize the spontaneous emission dynamics from the emission of dye molecules influenced by the substrates. For the R110 (R6G) dye molecule, the lifetime of the HMM1, HMM2 and reference samples are 1.576 (3.098), 2.893 (2.946) and 3.518 (4.102) ns, respectively, as shown in Fig. 3d,e). That is, the lifetime of the HMM1 for R110 (R6G) dye molecule is ~55% (25%) shorter than that of the reference sample, while the lifetime of the HMM2 for R110 (R6G) dye molecule is ~18% (28%) shorter than the reference sample. For the DCJTB dye molecule, Fig. 3f presents the lifetime of the HMM1, HMM2 and reference samples are 2.116, 3.251 and 4.812 ns, respectively. Thus, the lifetime of the HMM1 (HMM2) for DCJTB dye molecule is ~56% (32%) shorter than the reference sample. This reduced lifetime is attributed to the excitation of high-*k* modes that can increase the carrier recombination rate of the dye molecules. The enhancement of decay rate by assuming a dipole emitter on the top of HMMs substrate is:

3 $${{\rm{\Gamma }}}_{high-k}=\frac{{\mu }_{\perp }^{2}}{8\hslash {d}^{3}}\frac{2\sqrt{|{{\rm{\varepsilon }}}_{\parallel }|{{\rm{\varepsilon }}}_{\perp }}}{1+|{{\rm{\varepsilon }}}_{\parallel }|{{\rm{\varepsilon }}}_{\perp }},$$

where ${{\rm{\Gamma }}}_{high-k}$ is the additional decay rate only existing in the HMMs substrate because of the distinctive feature from the high-*k* modes, ${\mu }_{\perp }$ is the perpendicular dipole emitter and *d* is the distance of the dipole emitter of dye molecule away from the HMMs substrate^29^. Consequently, with considering this additional decay rate, the lifetime reduction of the dye molecules can be well understood.

### Numerical simulation

**Figure 4:**
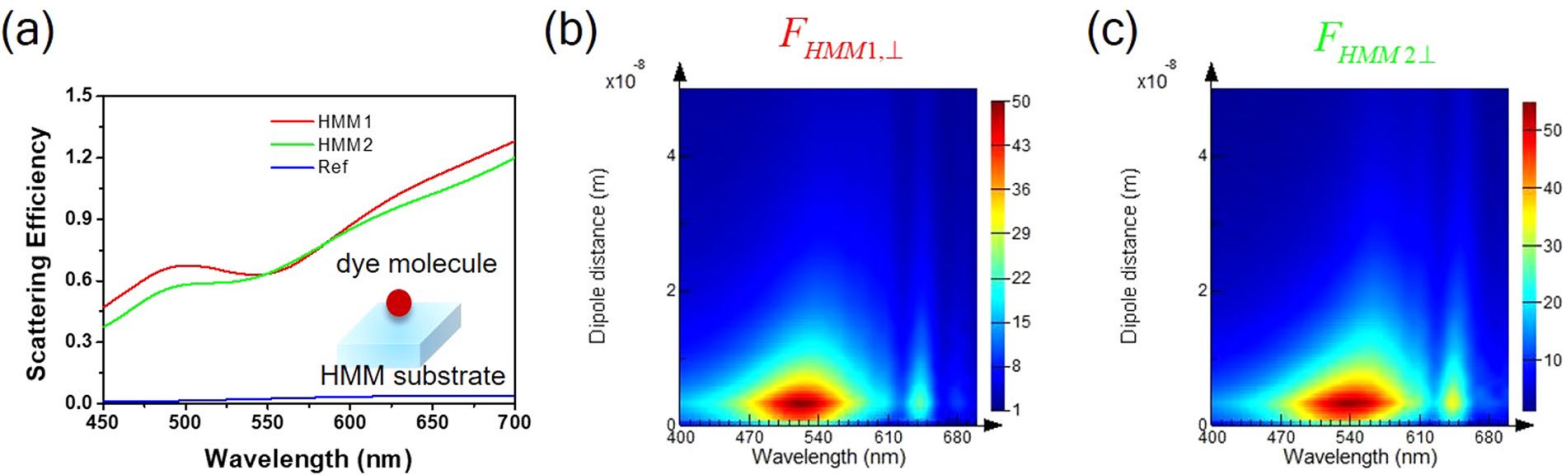
Theoretical analysis for scattering efficiency and Purcell factors. (**a**) The calculated scattering efficiency of the dye molecule with a sphere size (radius of 25 nm) and the refractive index is 1.33. (**b**) and (**c**) are the Purcell factors with a dipole emitter perpendicular to the HMM1 and HMM2 within 50 nm, respectively.

To stand out the notable effect from the transient HMMs substrates, we further examine the scattering efficiency $(\frac{{\sigma }_{scat}}{{A}_{scat}})$ as shown in Fig. 4a. The scattering cross-section (${\sigma }_{scat}$) is given by:

4 $${{\int }^{}}_{4\pi }\frac{|T{|}^{2}}{{k}^{2}|{E}_{i}{|}^{2}}d{\rm{\Omega }},$$

where *k* is the wave number and Ω is the solid angle^44^. $T={E}_{x}X+{E}_{y}Y$, where *X* (*Y*) is the scattering amplitude with *x* (*y*)-polarization of incident light. The scattering cross-sectional area (${A}_{scat}$) is the projective plane perpendicular to the incident light. Here, we use an effective dye molecule with a sphere size (radius of 25 nm) and the refractive index of 1.5. In general, the scattering efficiency of the HMM1 sample is larger than the HMM2 sample in the visible light, except in the wavelength region 545–585 nm. The scattering efficiency of the reference sample always stays below 0.03. The emission center of R110 (DCJTB) dye molecule is at 530 (650) nm, and the corresponding scattering efficiency for the HMM1 is 7.3% (7.9%) higher than the HMM2 sample. For the emission center of R6G dye molecule at 560 nm, the scattering efficiency for the HMM2 sample is 3.2% higher than the HMM1 sample. These scattering efficiencies also reflect the measured intensity of photoluminescence spectra. Figure S4 presents the distributions of far-field angular electric field intensity (|E|^2^) to understand the scattered field intensity under the normal incident of light, where *XY* (*XZ* and *YZ*) plane refers to the scattered direction parallel (perpendicular) to the substrate. With considering the HMM1 (HMM2) sample, Fig. S4a (S4d), S4b (S4e) and S4c (S4f) are the scattered |E|^2^ along *XY*, *XZ* and *YZ* planes, respectively, showing that the strong out-coupled intensity can be observed (*i.e*., the scattered |E|^2^ of the *XZ* and *YZ* planes are observed in the north sphere side). However, the scattered |E|^2^ of the reference sample is randomly distributed in all directions, representing the energy is dissipated to multiple angles rather than focuses on to the far-field direction as shown in Fig. S4g,h and i for *XY*, *XZ* and *YZ* planes, respectively.

To further explain the enhancement of photoluminescence intensity, here we calculated the Purcell factor to describe the enhancement rates influenced by the environment effect based on the spontaneous emission dynamics, which is composed of the dipole directions perpendicular (${F}_{\perp }$) and parallel (${F}_{\parallel }$) to the substrate determined by^45^:

5 $${F}_{iso}=\frac{1}{3}{F}_{\perp }+\frac{2}{3}{F}_{\parallel }.$$

Figures 4b,c are the Purcell factors with a dipole source above the surface within 50 nm perpendicular to substrates for the HMM1 and HMM2 samples spanning from wavelength 400–700 nm, respectively, while Fig. S5a,b are the dipole direction parallel to the substrates. Evidently, the Purcell factor reaches the highest at ~530 (560) nm for the HMM1 (HMM2) sample matching the measured photoluminescence intensity of the R110 (R6G) dye molecule as shown in Fig. 3a,b). However, for the R110 dye molecule on HMM2 sample (Fig. 3a), the emission intensity is not as high as expected based on the Purcell factor owning to the elliptical dispersion in this case. The purpose of the transient HMMs is primarily a proof of concept for the physics behind with the possible combination of HMMs and transient technology. To explain the fundamental working mechanism, we emphasize the excitation of high-*k* modes making the PDOS higher followed by increasing the transition rate. This increased transition rate leads to the enhancement of photoluminescence intensity as confirmed by the Fermi’s golden rule^46^.

### Dissolving configurations, demonstrating flexibility and integrating with optical components of the transient HMMs

**Figure 5:**
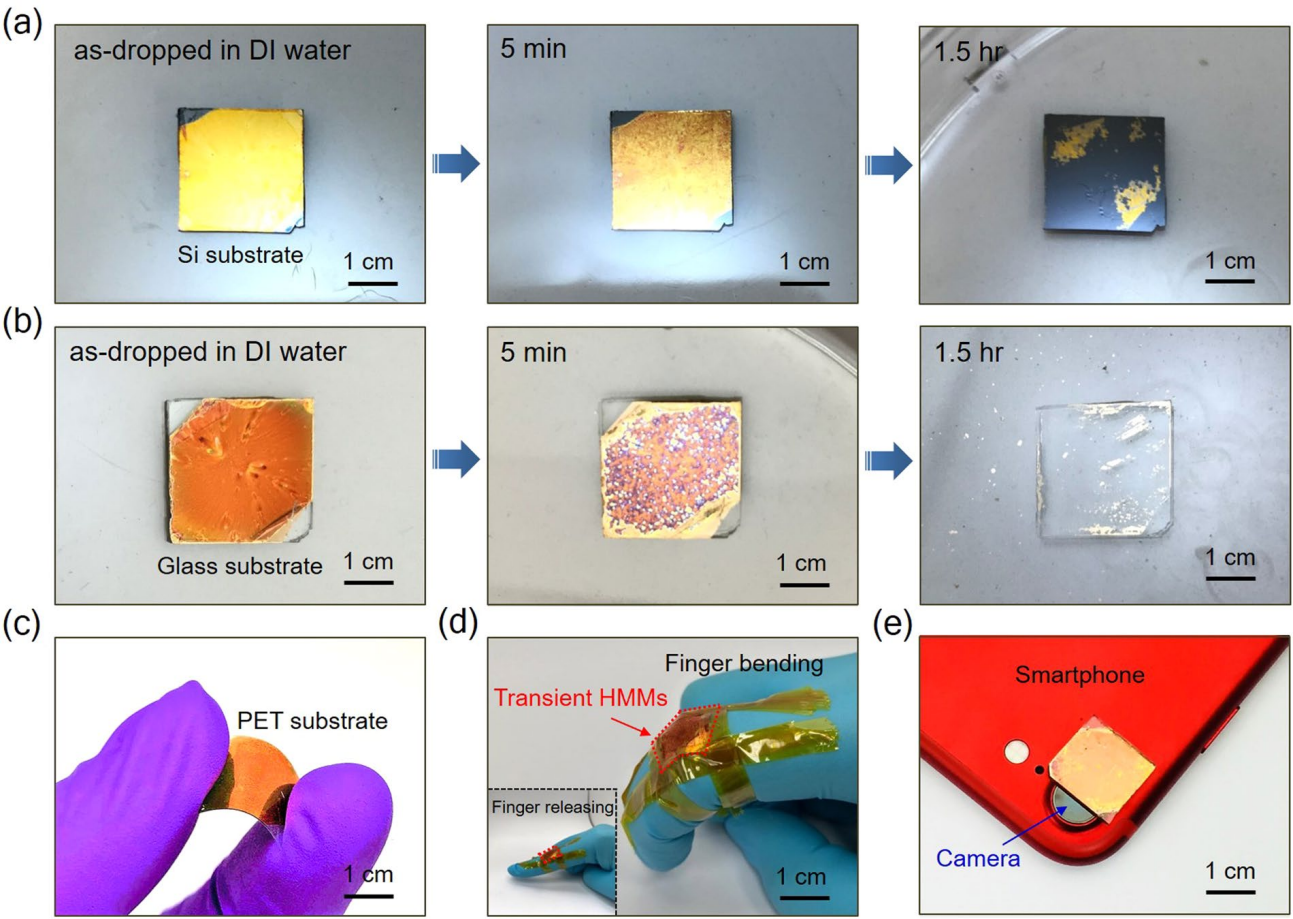
Demonstration of the dissolvability and the flexibility of the transient HMMs. (**a**) and (**b**) are the dissolving processes in deionized (DI) water for the HMM1 sample on Si substrate and HMM2 sample on glass substrate, respectively. (**c**) Transient HMMs on a PET substrate in a bending configuration. (**d**) Piece of a transient HMMs device was attached to a human finger in a bending gesture. Inset image is the finger in the releasing configuration. (**e**) Integration of the transient HMMs with a camera of smartphone.

As a proof of concept, Fig. 5a,b) is the optical image of the transient HMM1 (HMM2) on Si (glass) substrate during the dissolving process. Clear images can be seen that both the transient HMMs samples will be destroyed after putting them into DI water. After ~1.5 hr, both the transients HMMs almost disappear. More detailed dissolving process for the HMM1 and HMM2 are shown in Fig. S6a,b, respectively. Note that, these dissolving processes occur from the edge sides of the transient HMMs, then etching closer to the central part, and subsequently the multilayer structure is peeled off from the substrates resulting in the ultimately decomposed of the transient HMMs devices. To emphasize the importance of dissolving process can decrease or even destroy the unique properties of the HMMs, we consider the photoluminescence intensity of the R6G dye molecule on the HMM2 sample after immersing the substrates into DI water for 5 and 30 min as shown in Fig. S7. It indicates that the reduction of the emission intensity after immersing the HMM2 sample in 5 (30) min is ~63% (89%) as compared with the result shown in Fig. 3b. We even observe the emission intensity after immersing in 30 min is ~28% lower than the reference sample due to the quenching effect from the decomposed Au powders.

To further demonstrate the polymer-based transient HMMs can be used for future flexible and wearable optoelectronic devices, we fabricate the transient HMMs on a polyethylene terephthalate (PET) as shown in Fig. 5c. The flexible and wearable optoelectronic are important functionalities to broaden the applications of their natural behaviors^47–50^. The transient HMMs still remain their original functionalities rather than being cracked or damaged during the bending process. Figure 5d presents the integration of the transient HMMs onto a human finger as a wearable device with releasing and bending behavior. Owning to the lightweight and flexible characteristics, the angle between the releasing state and the bending state can reach approximately 90°, showing the outstanding mechanical tolerance property of the transient HMMs. Besides, the recent development in metalens has attracted tremendous attention, since it can be operated in the visible spectrum to focus light with only few hundred nanometers thick that can replace the normal lens including display, cameras and the consumer smartphone^51–54^. Therefore, we combine the transient HMMs with a consumer smartphone (Fig. 5e) as a degradable portable device that can minimize the waste for environmental friendly purpose. This combination broadens the usage of metalens device that can integrate with transient technology.

**Figure 6:**
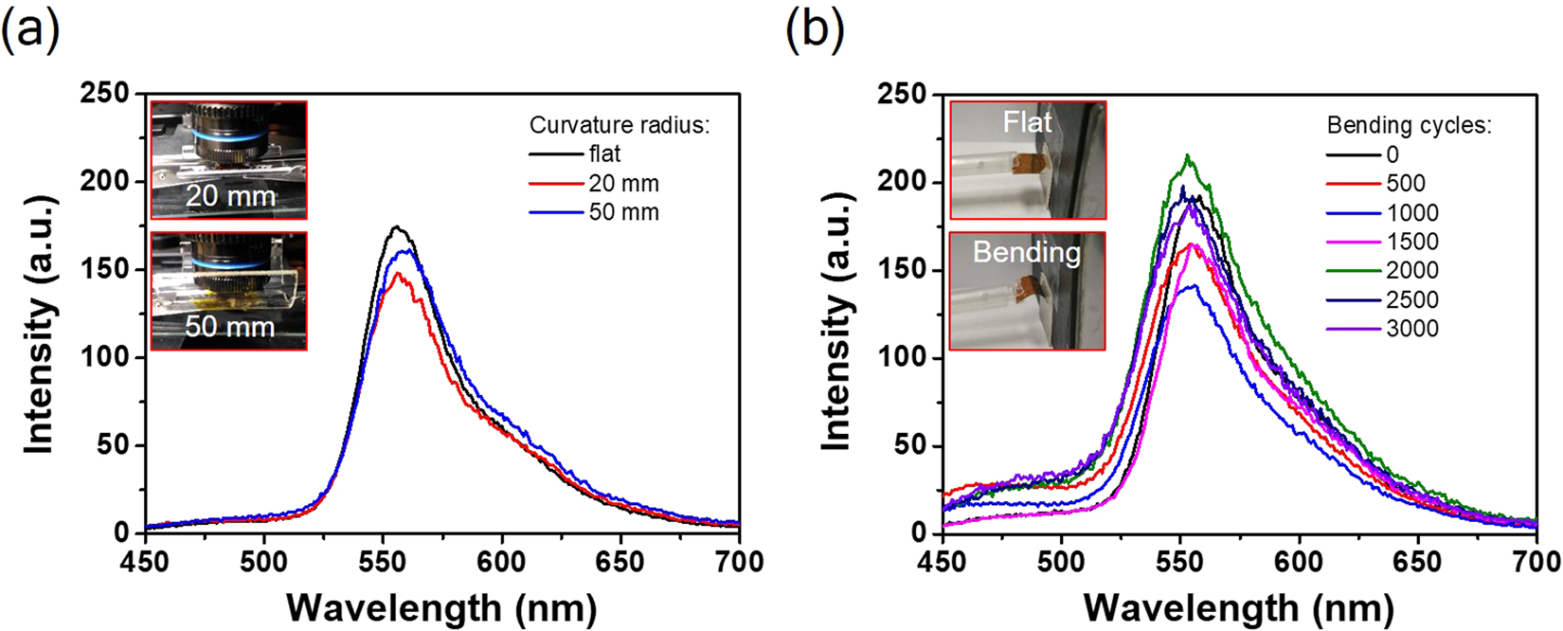
Bending performance of the transient HMMs on a flexible PET substrate. (**a**) Photoluminescence spectra of the R6G dye molecule on the HMM2 sample measured under different inner bending curvature radius. Insets are the curvature radius of 20 and 50 mm. (**b**) Photoluminescence spectra within 3000 bending cycles at a bending radius of ~5 mm. Insets are flat and bending behaviors of the transient HMMs device.

To demonstrate the functionality of the transient HMMs device after bending, we measured the photoluminescence intensities of the R6G dye molecule on the HMM2 sample with a PET substrate under different inner bending curvature radius (*e.g*., 20 and 50 mm) to test the bending performance as shown in Fig. 6a. Interestingly, the transient HMMs device shows the stable emission intensities without any particular photodegradation after 3000 bending cycles at a bending radius of ~5 mm as shown in Fig. 6b, illustrating the polymer-based HMMs device is suitable for flexible optoelectronics.

### Temperature effect on the transient HMMs

In terms of the real application, a temperature effect on the transient HMMs devices should be concerned. The working temperature is mainly determined by PVA, since it is a temperature-sensitive polymer. The suitable temperature for the transient HMMs devices is below its glass transition temperature (T_g_) 78–85 °C^55,56^. Because at temperatures above T_g_, the chains of PVA will have much more mobility and then reorient together with other chains, thus, the PVA behaves like a liquid. For a general concept of HMM, the multilayer structures should maintain in a flat thin film to provide the propagation of the high-*k* modes. Otherwise, the localized resonance from the metal clusters will dominate^57^.

### Outlook of the transient HMMs

Transient HMMs are promised to be the succeeding generation of optoelectronics with the following prospects. First, polymer-based transient HMMs are suitable for the flexible optoelectronics applications, such as electronic skin (e-skin), which is widely considered as the next generation technology that can integrate the human body seamlessly with robots as photochemical and photonics sensors. Second, the metal material from the transient HMMs devices can be recycled from the dissolved solution for the environmental friendly purpose. After using for a certain period of time, these devices can be easily dissolved just by putting into water. Furthermore, if the transient HMMs are made with bio-compatible materials, we can implant these devices into human body for future nanorobotics for *in-vitro* diagnostics or drug-delivery systems that can be triggered by the external light source. Moreover, owing to the flexibility and environmental friendly characteristics, transient HMMs are useful for the next generation photonic usages, such as anti-tamper technology, military camouflage and health-monitoring sensors with wearable devices.

## Conclusion

We have successfully made a first attempt to demonstrate the transient and flexible HMMs that are composed of PVA, a water-soluble polymer, and Au as multilayers. These multilayers show elliptical or hyperbolic dispersion with the precise design of multilayers compositions at different operating wavelengths, which can influence the varieties of emission enhancements from different dye molecules by tuning the PDOS. Simulation results from the scattering cross-section and the distributions of far-field angular electric field intensity demonstrate the out-coupling effect owing to the unique characteristics of HMMs. Besides, the calculations of the Purcell factors also show the high PDOS as confirmed by the measured spontaneous emission from the dye molecules. Furthermore, the dissolving process has been conducted with different immersion times in DI water. We found that the transient HMMs loss hyperbolic dispersion in 5 min and then totally disintegrated after ~1.5 hr. Owing to the environmental friendly compositions and flexibility of the transient HMMs, they possess a great potential for practical applications, such as wearable optoelectronics or degradable portable devices.

## Methods

### Fabrication of the transient HMMs

To prepare the transient HMMs, we used the Si wafers as the substrates in this experiments. The Si substrates (~1.5 × 1.5 cm) were ultrasonically cleaned for 20 min in acetone, ethanol and DI water in sequence to remove the absorbed contaminants. To further obtain higher quality Si substrates, we put them on a hotplate at 80 °C for 10 min to remove the unwanted moisture. To deposit the Au thin film, we put these Si substrates inside the chamber for thermal evaporation then deposited under a high vacuum condition (< 5 × 10^−7^ Torr). The deposition rate was remained at 0.5 Å/s. After that, we dissolved PVA into DI water at a mass fraction of 20 wt% and then stirred it for 1 hr at room temperature. Finally, it was sonicated for 1 hr to get a homogeneous mixture. The PVA solution was spin-coated on the samples at a rate of 2500 (3000) rpm to obtain the different thickness of 49 (42) nm. Afterwards, these samples were heated at 60 °C for 20 min to totally remove the DI water remained inside the PVA thin film. We repeated this procedure (PVA/Au pair) for 3 times to get the 4 pairs of multilayers structure. A recent report demonstrated that HMM structures with 4 pairs of multilayers were able to control the charge transfer dynamics of semiconductors^31^. The topmost layer of PVA also serves as a capping layer to avoid quenching effect from the emission of dye molecules^32^.

### Preparation of dye molecules

First, the R110 was dissolved into methanol at room temperature with a concentration of 1 mg mL^−1^ and then mixed with 8 mg mL^−1^ PVA. The solution was directly dropped on the transient HMMs devices and heated at 120 °C for 120 min. Second, 1 mg of R6G and 100 mg PVA were mixed together followed by dissolving in 10 mL DI water to prepare R6G. After that, we spin-coated the solution on the transient HMMs devices at a rate of 2000 rpm for 30 s. Third, the DCJTB was dissolved into dichloromethane (DCM) with a concentration of 3 mg mL^−1^ at room temperature. Afterwards, the solution was dropped-coated on the transient HMMs devices followed by baking them at 100 °C for 10 min on a hotplate.

### Characterization of the transient HMMs

We used the focused ion beam (FIB) system to characterize the geometry of the transient HMMs samples. To prepare the uniform cross-sectional slices from the transient HMMs samples, the FIB system was operated at a voltage of 30 kV and a current of 50 pA with Gallium ion source. Operating under such a low current is needed, since Gallium ion source possesses with a high energy that will destroy the multilayer components and make them mix together. Finally, the images were taken using FE-SEM with a tilted holder angle of 52°.

### Measurement of photoluminescence spectra and lifetime

The photoluminescence spectra were measured using a pulsed diode laser (Picoquant, PDL 800-B, center wavelength of 374 nm, 70 ps, 2.5 MHz), then these spectra were collected by a Horiba Jobin Yvon TRIAX 320 spectrometer. The lifetime measurement was carried out by a time corrected single photon counting (Pico Harp 300) system with the time resolution of 36 ps. All the photoluminescence dynamics were performed at room temperature.

### Numerical simulation

All simulation results were employed by the commercial electromagnetic software (Lumerical) under *x*-polarized normal illumination. The refractive index of PVA was set to be 1.48, dye molecule was 1.33 and Au was from Pelik^58–60^. Perfectly matched layers were used in the 3D directions to avoid the artificial numerical results from the boundary of computational region. We set the mesh size with 2 nm in the simulation results.

## Electronic supplementary material

Revised Supporting information
