# Supplementary material for: Transient and Flexible Hyperbolic Metamaterials on Freeform Surfaces
Source: Sci Rep. 2018 Jun 21;8:9469. doi: 10.1038/s41598-018-27812-4 (PMC6013475; doi:10.1038/s41598-018-27812-4)
Supplement: Supplementary file 1 — Revised Supporting information [file 41598_2018_27812_MOESM1_ESM.docx]

Supporting Information for

**Transient and Flexible Hyperbolic Metamaterials on Freeform Surfaces**

Hung-I Lin^1,2^, Kun-Ching Shen^3^, Shih-Yao Lin^2^, Golam Haider^2^, Yao-Hsuan Li^1^, Shu-Wei Chang^1^, and Yang-Fang Chen^1,2^*

^1^ Graduate Institute of Applied Physics, National Taiwan University, Taipei 106, Taiwan

^2^ Department of Physics, National Taiwan University, Taipei 106, Taiwan

^3^ Research Center for Applied Sciences, Academia Sinica, Taipei 115, Taiwan

*corresponding author: Yang-Fang Chen: [yfchen@phys.ntu.edu.tw](mailto:yfchen@phys.ntu.edu.tw)

1. **The calculation of Maxwell-Garnett theory.**

To understand the dispersion behaviors of layered structures, the corresponding effective dielectric tensors are provided and investigated. Since all the thickness of gold (Au) and poly(vinyl alcohol) (PVA) are much smaller than the operating wavelength, the effective permittivity tensors are assumed to be homogeneity and attributed to the effective medium theory (EMT). Thus, the dielectric tensors parallel ($\varepsilon_{\parallel}$) and perpendicular ($\varepsilon_{\perp}$) to the surface are given by:[^1^](#_ENREF_1)

$\varepsilon_{\parallel}=\varepsilon_{x}=\varepsilon_{y}=\sum_{n=1}^{2} f_{n}\varepsilon_{n}$,$\varepsilon_{\|}={f\varepsilon}_{\mathrm{Ag}}+{(1-f)\varepsilon}_{\mathrm{TiO}2}$ (S1)

$\varepsilon_{\perp}^{-1}=\varepsilon_{z}^{-1}=\sum_{n=1}^{2} f_{n}\varepsilon_{n}^{-1}$,$\varepsilon_{\text{┴}}=\frac{\varepsilon_{\mathrm{Ag}}\varepsilon_{\mathrm{TiO}2}}{{f\varepsilon}_{\mathrm{TiO}2}+{(1-f)\varepsilon}_{\mathrm{Ag}}}$ $\varepsilon_{\|}={f\varepsilon}_{\mathrm{Ag}}+{(1-f)\varepsilon}_{\mathrm{TiO}2}$ (S2)

$f_{n}=\frac{d_{n}}{\sum_{n=1}^{2} d_{n}}$, (S3)

where $\varepsilon_{n}$ is the permittivity and $d_{n}$ is the thickness of the $n$-the layer. The EMT calculation method can determine the hyperbolic dispersion (*i.e.*, $\varepsilon_{\perp}\cdot\varepsilon_{\parallel}<0$).[^1^](#_ENREF_1) Further, we used the Maxwell-Garnett theory to obtain the dispersion relation of the two HMM samples as shown in Fig. S1. The dielectric tensor components are given by:

$\varepsilon_{\perp}=\rho\varepsilon_{Au}+(1-\rho)\varepsilon_{PVA}$, (S4)

and

$\varepsilon_{\parallel}=\frac{\left( 1-\rho\right){\varepsilon_{Au}\varepsilon}_{PVA}+(1+\rho)\varepsilon_{PVA}^{2}}{(1-\rho)\varepsilon_{Au}+(1+\rho)\varepsilon_{PVA}}$, (S5)

where $\rho$ is the thickness of ﬁll fraction of Au in the stack of multilayer.

**Figure S1 |** (a) and (b) are the calculated effective dielectric tensors of the HMM1 and HMM2 samples, respectively. Light red (blue) regions are the wavelength range with hyperbolic (elliptical) dispersion. Black dashed line is the transition wavelength transforming from elliptical to hyperbolic dispersion, while for HMM1 (HMM2) is at 520 (560) nm.

1. **Iso-frequency curve of the transient HMMs.**

To provide the evidence of the existence of high-*k* modes from our proposed transient HMMs, where high-*k* modes can support the coupling effect in between the HMMs and the dye molecules instead of decaying as a form of evanescent field. Here we discuss the iso-frequency curves of the transient HMMs as shown in Fig. S2. The formula of iso-frequency curve can be written as:[^2^](#_ENREF_2)

${(\frac{k_{HMM,\perp}}{k_{0}})}^{2}\frac{1}{\varepsilon_{zz}}+{(\frac{k_{HMM,z}}{k_{0}})}^{2}\frac{1}{\varepsilon_{\perp}}=1$, (S6)

where $k_{0}=\omega/c$, while $k_{HMM,\perp}$ and $k_{HMM,z}$ represent the effective wave-vector of the perpendicular and parallel to the optical axis of HMMs substrates, respectively. Fundamentally, by properly controlling the metal/dielectric compositions of HMMs and operating under different wavelength, the iso-frequency curve changes from elliptic to hyperboloid. High-*k* modes occur when ${|k}_{HMM}|>{|k}_{air}|$. The criteria of wave passing through the multilayers-based HMMs (*e.g.*, HMM1 and HMM2) out-coupled to far-field requires to be side out-coupling, where the coupling interface of the HMM and air (*i.e.*, *yz* plane) also reaches the conservation of $k_{z}$ component (*i.e.*, $k_{HMM,z}=k_{air,z}$). This is due to the fact that side out-coupled energy from the high-*k* modes can be easily achieved by the multilayers design with Type Ⅱ dispersion (*i.e.*, $\mathrm{Re}\left( \varepsilon_{\perp} \right)<0$ and $\mathrm{Re}\left( \varepsilon_{zz} \right)>0$). Light can propagate from the HMMs to air with a refraction angle $\theta$, which we represent it in one quadrant for simplicity thus can be spread out to the whole plane geometry. Besides, this refraction angle between the refracted wave-vector and the normal line to the HMMs couple interface is given by: $\theta=\tan^{-1} ({k_{air,z}}/{k_{air,x}})=\tan^{-1} ({k_{HMM,z}}/{k_{air,x}})$. The iso-frequency curves behave similar Type Ⅱ dispersion by considering the HMM1 at 530 nm (Fig. S2a), 560 nm (Fig. S2b) and 650 nm (Fig. S2c), also 650 nm of the HMM2 sample, which means $\theta$ can range from 0° to 90°. On the other hand, the HMM2 sample at 530 nm (Fig. S2a) acts as EMM due to the fact that the shape of iso-frequency curve is elliptic, resulting in the limited wave-vectors modes. Furthermore, the iso-frequency curve of the HMM2 sample at 560 nm (Fig. S2b) is very sharp, which is close to a straight line. This will make the refraction from the HMM to air with angle almost ~0°.

**Figure S2 | Iso-frequency curves of the transient HMMs.** (a), (b) and (c) are the iso-frequency curves at 530, 560 and 650 nm, respectively, for all the samples used in this work. The bottom images are their corresponding wave-vectors out-coupling diagrams. The $\theta_{1}$ ($\theta_{2}$) is the maximum refraction angle of out-coupling wave-vector of the HMM1 (HMM2) sample.

1. **The calculation of effective permittivity.**

Figures S3a and S3b show the simulation results by three-dimensional (3D) finite-difference time-domain (FDTD) simulation to analyze the absorption performance of samples of the HMM1 and HMM2 samples, respectively. The absorption ($A$) spectrum is determined by: $A=100\%-T-R$, where $T$ and $R$ represent the transmission and reflection, respectively. The permeability remains unchanged because of not introducing any magnetic materials in this work$\mu=1$. Besides, to discuss the propagation behavior inside the layered structures, the effective refractive indices $(n+ik)$ can also be realized via $A$ property using the incoherent interference formula in a slab.[^3^](#_ENREF_3) It can be directly determined from the $T$ and $R$ spectra,

$n=\frac{(1+R_{ah})}{(1-R_{ah})}-\left[ \frac{4R_{ah}}{\left( 1-R_{ah} \right)^{2}}-k^{2} \right]^{1/2}$, (S7)

$n=\frac{(1+R_{\mathrm{ah}})}{(1-R_{\mathrm{ah}})}-\left[ \frac{4R_{\mathrm{ah}}}{\left( 1-R_{\mathrm{ah}} \right)^{2}}-k^{2} \right]^{1/2}$and $k=\frac{-\lambda}{4\pi t}ln\left\{ \frac{{\left[ T^{2}-{(1-R)}^{2} \right]+\left\{ \left[ T^{2}-{(1-R)}^{2} \right]^{2}+{4T}^{2} \right\}}^{1/2}}{2T} \right\}$, (S8)

where $R_{ah}=\frac{R}{1+\frac{{\left[ T^{2}-{(1-R)}^{2} \right]+\left\{ \left[ T^{2}-{(1-R)}^{2} \right]^{2}+{4T}^{2} \right\}}^{1/2}}{2}}$ , (S9)

where $R_{ah}$ is the surface reflectance between air and HMMs.

**Figure S3 | Optical properties of the transient HMMs.** (a) and (b) are the calculated transmission, reflection and absorption spectra of the HMM1 and HMM2 samples, respectively. (c) and (d) are the complex refractive indices, $n$ and $k$, derived from the transmission and reflection spectra. (e) and (f) are the effective permittivities, $\varepsilon_{y}^{eff}={(n+ik)}^{2}$, are subsequently determined.

1. **The calculation of the far-field angular electric field intensity (|E|^2^) distributions.**

To further develop the scattering field intensity, Fig. S4 depicts the far-field angular |E|^2^ distributions with the normally incident of light from 450 nm to 700 nm. For the HMM1 (HMM2 and reference sample), Figs. S4a (S4d and S4g), S4b (S4e and S4h) and S4c (S4f and S4i) are the scattered |E|^2^ along *XY*, *XZ* and *YZ* planes, respectively. The north sphere (*i.e.*, Phi below 3.14 (rad)) represents the scattered |E|^2^ out-coupled to the far-field rather than being trapped inside the multilayers structures, while the south sphere is the scattered |E|^2^

**Figure S4 | Far-field angular |E|^2^ distributions.** (a) ((d) and (g)), (b) ((e) and (h)) and (c) ((f) and (i)) are the scattered |E|^2^ along *XY*, *XZ* and *YZ* planes, respectively, of the HMM1 (HMM2 and reference sample).

1. **The calculation of Purcell factors in parallel dipole emission.**

Figure S5 presents the Purcell factors within 50 nm with a dipole emitter parallel to the transient HMMs substrates.

**Figure S5 | Theoretical analysis of Purcell factors.** (a) and (b) are the Purcell factors within 50 nm with a dipole emitter parallel to the HMM1 and HMM2, respectively.

1. **Discussion of Fermi’s golden rule.**

In quantum mechanism, Fermi’s golden rule explains the transition rate $(\Gamma_{fi})$ from the initial state $(\Psi_{i})$ to the final state $(\Psi_{f})$:[^4^](#_ENREF_4)

$\Gamma_{fi}=\frac{2\pi}{\hbar}{\left| <\Psi_{f} \right|H^{'}\left| \Psi_{i}> \right|}^{2}\rho(k)$, (S10)

where $\hbar$ is the reduced Planck constant, $\rho(k)$ is the density of state, which is proportional to the wavevector, *i.e.*, $\rho(k)\propto k^{3}$. $<\Psi_{f}|H^{'}|\Psi_{i}>$ is the inner product perturbation Hamiltonian ($H^{'}$) between $\Psi_{i}$ and $\Psi_{f}$ in a form of matrix element. Consequently, these unique transition rate makes HMMs possess with higher emission intensity than the isotropic medium materials.

1. **Dissolvability of the transient HMMs.**

Figures S6a and S6b are the optical images of the detailed dissolving processes in deionized (DI) water of the HMM1 sample on silicon (Si) substrate and HMM2 sample on glass substrate within 1.5 hr, respectively. We can observe that the etching process starts from the edge side and then moves to the center part, finally the whole multilayers structures is peeled off.


**Figure S6 | The detailed dissolving processes of the transient HMMs.** (a) and (b) are the detailed dissolving process in DI water within 1.5 hr of the HMM1 sample on Si substrate and HMM2 sample on glass substrate, respectively. All the scale bar is 1 cm.

1. **The reduction of the photoluminescence intensity of transient HMMs.**

Figure S7 shows the photoluminescence intensity of the R6G dye molecule on the HMM2 sample after immersing the substrate into DI water for 5 and 30 min, measured by a 374 nm pulsed diode laser at the pumping energy density of 103 μJ/cm^2^ at room temperature.

**Figure S7 | Photoluminescence spectra after dissolving the transient HMMs substrate into DI water.**

**References**

R1 Cortes, C. L., Newman, W., Molesky, S. & Jacob, Z. Quantum nanophotonics using hyperbolic metamaterials. *J. Opt.* **14**, 063001 (2012).

R2 Ferrari, L., Smalley, J. S. T., Fainman, Y. & Liu, Z. Hyperbolic metamaterials for dispersion-assisted directional light emission. *Nanoscale* **9**, 9034-9048 (2017).

R3 Gao, J. *et al.* Experimental realization of epsilon-near-zero metamaterial slabs with metal-dielectric multilayers. *Appl. Phys. Lett.* **103**, 051111 (2013).

R4 Dowling, J. P. Spontaneous emission in cavities: How much more classical can you get? *Found. Phys.* **23**, 895-905 (1993).
